# Herbivore and detritivore effects on rainforest plant production are altered by disturbance

**DOI:** 10.1002/ece3.5316

**Published:** 2019-06-04

**Authors:** Chelse M. Prather, Gary E. Belovsky

**Affiliations:** ^1^ Department of Biological Sciences University of Notre Dame Notre Dame Indiana; ^2^ Department of Biology University of Dayton Dayton Ohio

**Keywords:** decomposer, ecosystem process, gastropod, light gap, phasmid, plant growth, rainforest

## Abstract

Consumer effects on rainforest primary production are often considered negligible because herbivores and macrodetritivores usually consume a small fraction of annual plant and litter production, even though consumers are known to have effects on plant production and composition in nontropical systems. Disturbances, such as treefall gaps, however, often increase resources to understory food webs, thereby increasing herbivory and feeding rates of detritivores. This increase in consumption could lead to more prominent ecosystem‐level effects of consumers after disturbances, such as storms that cause light gaps. We determined how the effects of invertebrate herbivores (walking sticks) and detritivores (litter snails) on understory plant growth may be altered by disturbances in a Puerto Rican rainforest using an enclosure experiment. Consumers had significant effects on plant growth, but only in light gaps. Specifically, herbivores increased plant growth by 60%, and there was a trend for detritivores to reduce plant growth. Additionally, plant biomass tended to be 50% higher with both consumers in combination, suggesting that herbivores may mediate the effects of detritivores by altering the resources available to detritivore food webs. This study demonstrates that disturbance alters the effects of rainforest consumers, and, furthermore, that consumer activity has the potential to change rainforest successional processes.

## 1 INTRODUCTION

Disturbances drive plant growth and plant community structure in many different ecosystems, including highly productive tropical forests, where, for example, an increase in light underneath a treefall gap can increase plant growth and alter plant community structure (Brokaw & Grear, 1991). Beyond primary producers, higher trophic levels are influenced by disturbances as well. Herbivores in many different ecosystems respond to changes in plant communities from disturbances in a variety of ways, and their taxonomy or feeding guild may affect their responses (Lewinsohn, Novotny, & Basset, 2005). Light gaps in rainforests promote the rapid growth of new leaves which increases herbivory (Angulo‐Sandoval & Aide, 2000; Spiller & Agrawal, 2003), abundances of gap‐specialist plants often preferred by herbivores (Coley & Barone, 1996), and the litterfall reaching the forest floor (Lodge & McDowell, 1991). Thus, consumer effects on plants may be amplified in light gaps where both herbivores and detritivores have greater resources available when consumers are limited by food. Consumer effects on vegetation structure may be modified by other factors, such as season (highly seasonal pine systems: Classen, Overby, Hart, Koch, & Whitham, 2007), but, to our knowledge, disturbance alterations of consumer effects on ecosystems have not been shown. In this study, we use a manipulative enclosure experiment to determine whether the effects of consumers on aboveground and belowground plant growth in a Puerto Rican rainforest are altered by disturbance.

Compared to other factors such as disturbances, the effects of rainforest consumers on ecosystem processes have not been well studied for several reasons. First, the low biomass of consumers relative to primary producers has led to a pervasive view that consumers may not significantly alter plant production (Feeley & Terborgh, 2005). Additionally, selecting focal consumers from these diverse, complicated, and often unknown tropical food webs is difficult. Finally, the overwhelming effects of disturbances may easily confound consumer effects, which can make designing effective studies more difficult. All of these factors have led consumer effects on rainforest plants to be understudied.

Studies in nontropical systems have shown that herbivores and macrodetritivores may affect plant available nutrients and plant communities through several different mechanisms. First, consumer feeding on plants and litter can change the quality and quantity of plants and litter. Herbivores negatively affect aboveground plant biomass by directly feeding on leaves, but foliage loss to herbivores may stimulate plants to reallocate biomass belowground (Dyer, 1993). Herbivory may also stimulate plants to produce secondary compounds, and this effect may be especially important in tropical tree–insect systems (Coley & Barone, 1996). Macrodetritivores that directly feed on litter or litter microbes can also affect nutrients available to plants (Gonzalez & Seastedt, 2001; Wang, Ruan, & Han, 2010). These effects can come through their assimilation of litter or through their feeding on litter; decomposers can accelerate the release of plant‐available nutrients by accelerating decomposition through their litter comminution, which exposes more surface area to litter microbial activity, including tropical forests (Gonzalez & Seastedt, 2001).

Secondly, the production of excrement by consumers can also affect plant production. Herbivore and detritivore excrement production may either stimulate by providing nutrients easily taken up by plants (Fonte & Schowalter, 2005; Frost & Hunter, 2004; Rinker, Lowman, Hunter, Schowalter, & Fonte, 2001; Sirotnak & Huntly, 2000) or inhibit nutrient availability if microbes readily colonize frass and take up these nutrients (Lovett & Ruesink, 1995).

Lastly, selective feeding of consumers on plants and litter or microbes can change the functional composition of the plant and litter microbial communities (Weisser & Siemann, 2004). Selective feeding by herbivores may cause a shift in plant community composition, which in turn either increases or decreases nutrient release from litter depending on the nutrient content of the preferred plant species. For example, when herbivores prefer highly palatable, fast decomposing plants, plants that are less palatable and slower decomposing increase in abundance (Pastor, Naiman, Dewey, & McInnes, 1988). Decomposers respond negatively to the influx of poor‐quality litter produced by the abundant slow decomposing plants, consequently reducing decomposition and the rate at which nutrients are available to plants, thereby decreasing primary production (Belovsky & Slade, 2002; Brown & Gange, 1992; Feeley & Terborgh, 2005; de Mazancourt & Loreau, 2000; Pastor et al., 1988). Alternatively, herbivores selectively feeding on slower‐decomposing plants may increase primary production by increasing high‐quality resources to decomposers and ultimately plant nutrient availability (Belovsky & Slade, 2000, 2002; Holland, 1995; McNaughton, 1985). Macrodetritivore selective feeding on litter microbes may alter nutrient availability, but this mechanism has rarely been explored in terrestrial systems (Moore, Walter, & Hunt, 1988; Wardle, Bonner, & Barker, 2002). Depending on the functional role of the preferred microbial group, selective litter feeding or microbivory may increase or decrease plant available nutrients.

The combined effects of herbivores and macrodetritivore on vegetation structure may either enhance or diminish the singular effects of each group (Wardle & Bardgett, 2005). In some systems, the facilitation of nutrient release by macrodetritivores allows plants to compensate for biomass lost through herbivory (Brody, Palmer, Fox‐Dobbs, & Doak, 2010; Poveda, Steffan‐Dewenter, Scheu, & Tscharntke, 2005). Alternatively, because selective feeding by herbivores can either increase or decrease the quality of resources provided to decomposer communities, decomposition rates may either increase or decrease depending on an herbivore's feeding preference (as described above), thereby resulting in positive or negative feedbacks to plants and herbivores. The combined effects of multiple consumer trophic groups have not been studied in tropical forests.

These mechanisms of consumer impacts on plant production act concurrently; thus, in this study, we measure the net effects of consumer presence on plant production. We used abundant generalist consumers, walking sticks (*Lamponius portoricensis* Rehn) and litter snails (*Megalomastoma croceum* Gmelin), to represent herbivore and macrodetritivore groups, respectively. We used an enclosure experiment to manipulate consumers in open and closed canopy sites and measured their effects to litter quantity and quality, and aboveground and belowground plant growth. Overall, we predicted that the effects of both consumers would be amplified in light gaps because of increased plant growth and litterfall in disturbed sites. Specifically, we predicted that focal herbivores that prefer to consume faster decomposing plants (Sandlin & Willig, 1993; C. Prather, unpublished data) altering litter quantity and quality and thereby reducing primary production, and that detritivores would increase primary production by increasing nutrient availability to plants through litter comminution. We also predicted that the herbivore's effects on plant community composition through selective feeding would limit any stimulating effects of detritivores by decreasing quality of the resources reaching the detrital food web. This study is part of a larger study of these consumers’ effects on other ecosystem processes, including nutrient cycling and decomposition (Prather, 2011).

## 2 METHODS

This study was conducted at the Luquillo Long‐Term Ecological Research site (Luquillo LTER), located in the Northeastern corner of Puerto Rico (18°10′N, 65°30′W). Luquillo is a subtropical montane wet forest, growing on deep Oxisols and Ultisols of the Zarzal clay series, which receives approximately 3,500 mm of rain annually (Waide & Reagan, 1996). This study was conducted at around 300 m above sea level in the Tabonuco forest, named for the dominant tree, Tabonuco (*Dacroydes excelsa* Vahl). Luquillo is in a constant state of secondary succession because the forest is frequently hit by tropical storms (Waide & Lugo, 1992). This insular forest has a relatively low floral and faunal richness compared with mainland tropical sites, and thus, Luquillo is one of the only tropical forests where the food web has been described in detail (Waide & Reagan, 1996). This forest is, therefore, an ideal location to determine the role of consumers in rainforests.

**Figure 1:**
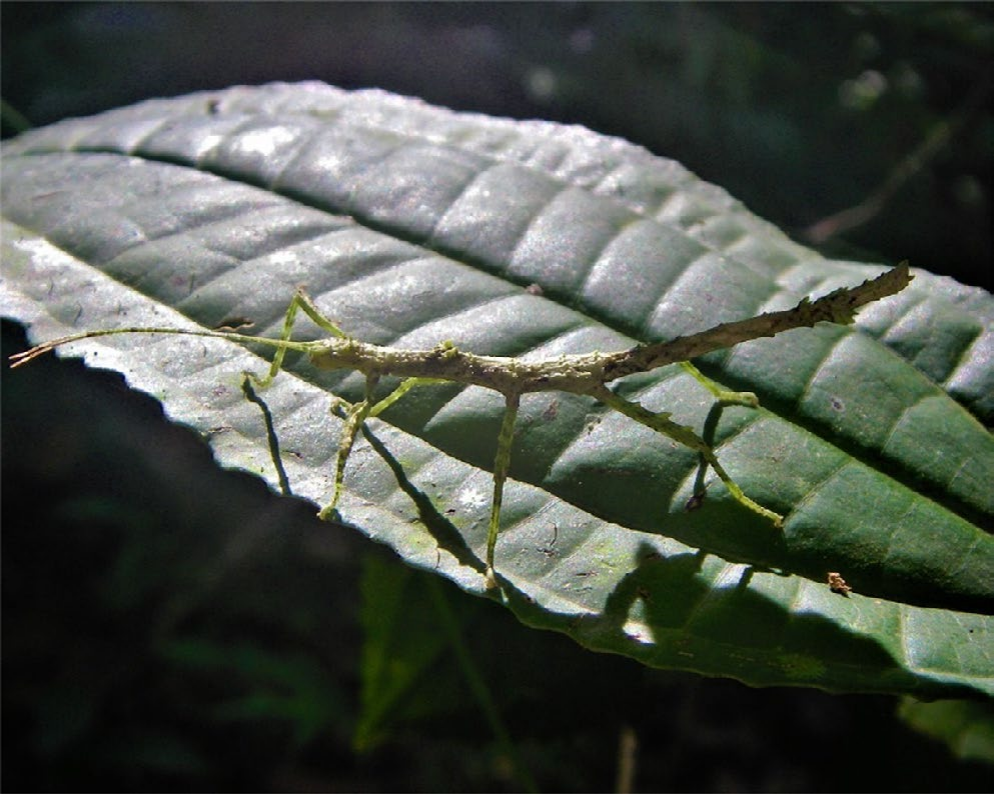
*Lamponius porticensis* juvenile on a *Miconia prasina* leaf

The methods for this experiment are described in detail elsewhere (Prather, Belovsky, Cantrell, & González, 2018). Focal plants and consumers were chosen for this study because they are abundant in the understory, commonly studied, and easy to transport and logistically manipulate. *Miconia prasina* (SW) DC. (grandillo bobo) and *Piper glabrescens* (Miq) C. DC. (Guyanese pepper) are abundant members of the understory plant community. The genera *Miconia* (Melastomataceae) and *Piper* (Piperaceae) are extremely speciose in the Neotropics with 19 and 12 species in the Caribbean, respectively (Molina & Alemany, 1997). Species of these genera have been studied together in several Neotropical rainforests (Baldwin & Schultz, 1988; Denslow, Vitousek, & Schultz, 1987). *Miconia prasina* is a shrub‐like tree that is an important early colonizer at Luquillo (Aide, Zimmerman, Rosario, & Marcano, 1996). *Piper glabrescens* is a common understory shrub that is relatively faster decomposing (~35% faster) than *M. prasina* (Prather, 2011; Prather et al., 2018). The focal invertebrate consumers in this experiment were *Megalomastoma croceum*, which is the most abundant litter snail at Luquillo (Prather, 2011) and *Lamponius portoricensis* Rehn (Figure 1), which is the most abundant generalist herbivore at Luquillo (Willig, Sandlin, & Gannon, 1993), whose effects on decomposition rates and plant available nutrients have been studied (Fonte & Schowalter, 2005).

Enclosures are commonly used to test the ecosystem‐level effects of consumers (Schmitz, 2004). This enclosure experiment was a fully crossed, 2 × 2 × 2 factorial design, manipulating herbivore, detritivore, and canopy cover presence (light gaps and closed canopy sites). We used un‐enclosed control plots to test the effect of the enclosures themselves. We replicated enclosures and controls (*n* = 3) in light gaps (recently disturbed prior to the experiment for the maintenance of power lines that run through the forest, <10% canopy cover) and closed canopy sites (>90% canopy cover), which were located in close proximity to one another (<500 m) on similar vegetation types for a total of 24 enclosures and 6 controls.

Mesh enclosures (0.15 mm from BioQuip) were supported by a PVC frame (1.6 m × 1.6 m), and trenched >30 cm below the ground to keep out soil organisms. After construction, all litter and visible organisms (including plants) were removed from inside enclosures and controls, and nonfocal plants and macroorganisms were removed during the experiment. Litter pooled from a common source (1,050 ± 50 g, the average amount collected from 36–1.6 m × 1.6 m areas near the study sites) was added to plots to initially create a similar litter layer across plots (the chemical composition of this litter, and of litter and green leaves from the plant species used can be found in Prather et al., 2018).

Individuals of each plant species (0.35–0.75 m tall) were transplanted into seedling bags and grown for 3 months under similar light conditions in rainforest soil pooled and homogenized from one site. Ten understory plants (five individuals of each species) were randomly chosen for each enclosure and control. This is a naturally occurring density of understory plants in this forest. To allow for easier extraction of belowground biomass, individuals were planted in PVC pipes (10.16 cm diameter, ≈0.25 m tall) with holes to allow the exchange of nutrients and water with the soil. Plants were watered with rainwater collected near plots for three days after planting and left to establish. All consumers were stocked in treatment enclosures at natural abundances: ≈3.6 fresh g of walking sticks per treatment (≈1.8 g/m^2^, six individuals: two adult males, one adult female, two juveniles, and one nymph individual) and ≈11.4 fresh g of snail per enclosure (≈5.7 g/m^2^; nine individuals across a range of size classes; see Prather, 2011 for sampling methods). This biomass of walking sticks and snails is proportionally equivalent to ≈5% of plant biomass and 1% litter biomass initially in enclosures, respectively, when animal weights are converted to dry biomass. For treatments with both consumers, both consumers were added at the above biomasses. Consumers were collected from the field and stored overnight in aerated tupperware containers before stocking in enclosures over 2 weeks in August of 2005. Thereafter, herbivores and detritivores in treatments were sampled every 4 months and initial treatment biomass was held constant (i.e., mortality was compensated with fresh animals, and new births were removed from experiments if this brought the treatment over the initial biomass).

The number, length, and width of stems, leaves, branches, and reproductive parts were measured on each individual plant after transplantation into study plots (August, 2005) and annually thereafter in the dry season at Luquillo (January of 2006–2008). Plant abundances were held constant by removing any new seedlings of focal plants or nontarget plants and replacing any dead plants. All plants were harvested at the end of the experiment in August of 2008, and plants were dried for at least 48 hr at 60°C until reaching a constant weight. Dry biomass of constituent plant parts was used to obtain allometric relationships to estimate biomass of individuals for each year (see Appendix S2). We were not able to extract the total belowground biomass because significant root mass grew through the holes in pipes that allowed the exchange of nutrients and water, but any differences in roots growing outside these pipes could not be easily measured. Therefore, to estimate belowground biomass, we extracted the total amount of soil and roots from pipes (10.16 cm diameter, ≈0.25 m tall), carefully removed the roots from soil, and scaled the root biomass by the average amount of soil in each pipe (800 g; Prather, 2011). To determine the relative abundance of different litter types in each enclosure and control, once annually (May, 2006–2008), all litter was carefully removed from the plots, sorted, weighed, and put back into the experiment.

Any data that violated assumptions for parametric tests were transformed using appropriate transformations. All statistical analyses were completed with SAS 9.4. *p*‐Values < 0.05 were considered significant. Results from allometric relationships were used to show growth of plants over time (Appendix S2). We used fully crossed, fixed‐effects ANOVAs with four main factors (canopy cover presence, herbivores and detritivores present, and plant species) to analyze treatment effects on final plant biomass (the biomass of all plant individuals of each species in a plot), final belowground biomass, and the arcsine‐transformed ratio of aboveground: belowground biomass. To further illustrate potential interaction differences between consumer treatments, we calculated the percent difference between each consumer treatment plant biomass (herbivore present, detritivore present, and both consumers present) and plant biomass with no consumers present (total exclusion enclosures) at each time period. We compared control plots (enclosure absent) to the herbivore + detritivore enclosures (enclosure present) using nested 2 × 2 ANOVAs (enclosure present, canopy cover present, and species nested within enclosure) to determine the effect of enclosure presence on each response variable. Enclosures with both consumers should most closely represent the whole forest (but with the enclosure present) since consumer treatments consisted of a natural abundance of organisms.

## 3 RESULTS

**Figure 2:**
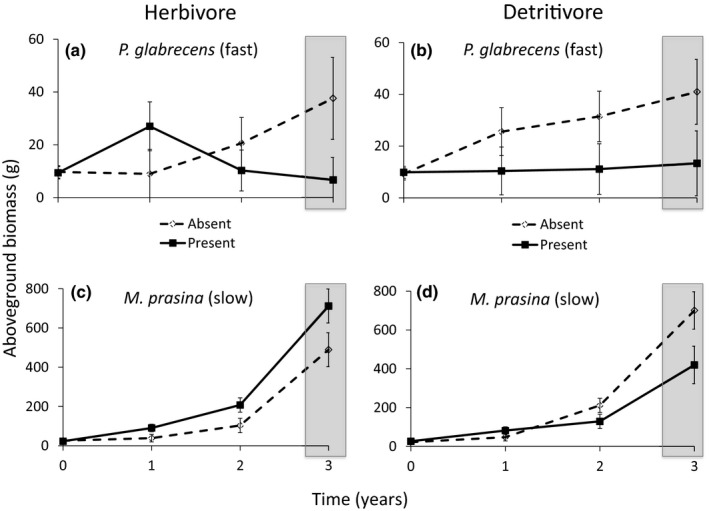
Consumer effects on biomass of each plant species in light gaps over time. Dashed lines represent consumer absence, and solid lines indicate consumer presence. Top two panels are herbivore (a) and detritivore (b) effects on *Piper glabrescens *biomass, and bottom two panels are herbivore (c) and detritivore (d) effects on *Miconia prasina* biomass. “Fast” refers to *P. glabrescens* litter decomposing faster than *M. prasina* litter (“Slow”). ANOVA tests were conducted on the final time point (shaded gray)

*Miconia prasina* individuals grew ~90% larger and had larger root masses (plant species, *df* = 1,32, *F* = 6.20, *p* = 0.014) than *P. glabrescens* individuals across all treatments (Figure 2). As expected, both plant species were smaller in closed canopy sites, leading to an order of magnitude difference between the light gaps and closed canopy sites at final harvest (canopy cover, *df* = 1,32, *F* = 572.15, *p* > 0.001). Greater biomass of both plant species was stored belowground in closed canopy sites (aboveground:belowground biomass—0.467 ± 0.15) compared to light gaps (1.268 ± 0.18; Appendix S1: Figure S1). Enclosures had few significant effects on plants: *P. glabrescens* individuals were significantly smaller inside enclosures (≈45% smaller inside enclosures than outside; enclosure * species, *df* = 1,12, *F* = 3.46, *p* = 0.044), but enclosures did not affect *M. prasina*.

**Figure 3:**
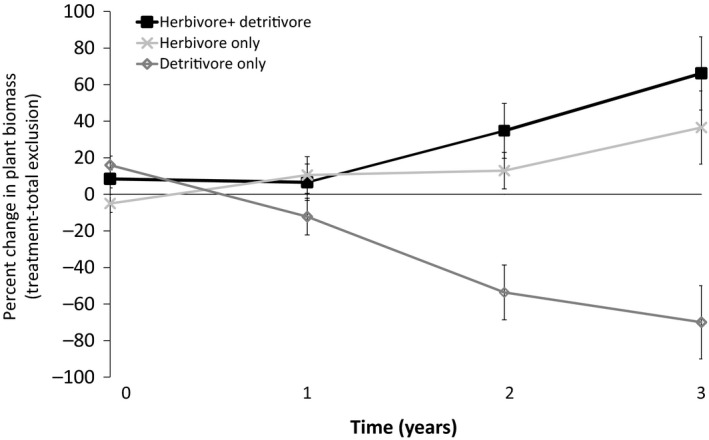
Treatment effects on total plant aboveground biomass (percent change between treatment biomass and total exclusion enclosures)

Both herbivores and detritivores tended to alter patterns of aboveground plant biomass, but *only* in light gaps (Figure 2; herbivore * canopy cover * plant species: *df* = 1,32, *F* = 6.348, *p* = 0.02; detritivore * canopy cover * plant species: *df* = 1,32, *F* = 7.20, *p* = 0.17). Neither consumer had significant effects on aboveground or belowground plant biomass in closed canopy sites, so below we only describe results from light gaps. Although herbivores had no significant effects on belowground biomass, aboveground *M. prasina* biomass nearly doubled with herbivores present (from 552 g without herbivores to 1,080 g with herbivores) and *P. glabrescens* biomass decreased by about 25% (from 370 g with herbivores to 301 g without herbivores; herbivore * canopy cover * plant species: *df* = 1,32, *F* = 6.348, *p* = 0.02). There was a trend for aboveground biomass of both plant species to decrease with detritivores present: *P. glabrescens* biomass decreased by 74% by the end of the experiment (from 371 g to 90 g with detritivores; detritivore * canopy cover * plant species: *df* = 1,32, *F* = 7.20, *p* = 0.17; Figure 3). Also, both plant species stored more biomass belowground with detritivores present (Appendix S1: Figure S1). We found no significant interactions between consumers using ANOVA. However, the percent change in plant biomass between treatments with and without detritivores was significantly lower than herbivore only or herbivore + detritivore treatments (Figure 3). This result suggests a nonadditive interaction between consumers, where herbivores mediate detritivore effects.

## 4 DISCUSSION

This study is one of the first to demonstrate that invertebrate consumers can alter plant growth and composition in a rainforest similar to their nontropical counterparts (see also Feeley & Terborgh, 2005). Although this finding is not surprising, given that we do not expect the mechanisms of consumer effects to be different between tropical and nontropical systems, it is important because the effects of consumers on plant communities in tropical systems are not well‐studied and often assumed to be negligible. In addition, not only were consumer effects amplified by disturbance as we predicted, consumers in this forest *only* affected plants in light gaps. This finding indicates that consumers may strongly affect rainforest successional processes, a particularly important finding for the Luquillo forest because of its disturbance‐driven dynamics (Waide & Lugo, 1992). Herbivore control over successional processes has been shown in other highly dynamic systems (e.g., intertidal zones—Lubchenco, 1983; temperate old fields and woodlands—Brown & Gange, 1992). Herbivores conspicuously mediated the effects of detritivores in this forest. This finding has important implications for rainforest functioning, where it is commonly thought that detritally derived nutrients predominately control primary production.

We observed that herbivore consumption promoted a less palatable, slower‐decomposing plant community in this forest in light gaps. The effect of herbivores having effects in light gaps is not novel: certain herbivores, including this species (Willig et al., 1993), have been shown preference for light gaps and early successional species of plants (Coley & Barone, 1996). This nocturnal species has a preference for certain species that are often more prevalent after treefalls (e.g., *P. glabrescens*), and thus is often associated with these habitats. Our original prediction that herbivores selectively feeding on fast decomposing plants would reduce aboveground plant growth, however, was based upon an assumption that our two focal plant species had similar foliar nutrient contents. In contrast, chemical analyses show that *P. glabrescens* leaves have significantly 40% higher foliar nitrogen than *M. prasina* (Prather et al., 2018). Nitrogen released from nitrogen‐rich *P. glabrecens* tissue by herbivory allows total primary production to increase, at least in the short term. In line with the finding that these herbivores produce increases in nutrient availability, *L. portoricensis* has been shown to increase decomposition and available nutrients in a previous short‐term study (Fonte & Schowalter, 2005; Schowalter, Fonte, Geagan, & Wang, 2011). These nutrients allow nitrogen‐poor *M. prasina* plants to build greater amounts of plant tissue per unit of nitrogen that they acquire, thus increasing overall plant production, and this increase in woody plant growth would be consistent with a nutrient pulse that would accompany a litter pulse (Wood, Lawrence, Clark, & Chazdon, 2009), such as that which might accompany an herbivory event.

This increase in plant production with herbivory is, however, likely short‐term. A shift to a lower quality, less palatable plant community produces low‐quality resources (litter) for decomposer food webs, thus decreasing decomposition rates and ultimately reducing nutrients available to the plant community. These changes likely occur on a longer time scale than this experiment ran. In order to see a reduction in primary production, the experiment would need to run until herbivory decreased the biomass of preferred host plants to low enough levels to induce an herbivore feeding switch to slower‐decomposing plants. Shifts to less palatable plants communities have been shown to decrease nutrient availability and reduce primary production over time in other systems (Belovsky & Slade, 2002; Feeley & Terborgh, 2005; de Mazancourt & Loreau, 2000; Pastor & Naiman, 1992). These predictions, though, could be augmented by the presence of other organisms: for instance, the presence of mat‐forming basidiomycetes that are able to break down poor‐quality litter, especially in low N environments, could allow a less palatable plant community to continue to be productive (Lodge et al., 2008).

Although not statistically significant, detritivore effects to plants tended to be greater in light gaps, likely because the reduced litter produced in plants limited by light in closed canopy sites reduced snail activity, and thus their effects on ecosystem processes. These trends of detritivore alterations of plant growth in disturbed sites were not surprising since it is thought that tropical production is primarily driven by detritially derived nutrients. (Waide & Reagan, 1996). However, that these detritivore‐induced trends tended to be a *reduction* of aboveground biomass was unexpected; we predicted that detritivores would increase primary production by increasing nutrient availability to plants through litter comminution. Detritivores have been experimentally shown to reduce plant biomass in other ecosystems (collembola in old fields—Scheu, Theenhaus, & Jones, 1999); however, this reduction was due to a microbivorous species, which largely reduced root biomass. In our study, detritivores increased belowground biomass (Appendix S1: Figure S1), indicating a potential decline in soil nutrients if plants are utilizing greater root mass to more efficiently acquire soil nutrients at low concentrations. In fact, total soil N concentration decreased by about 45% with detritivores present (Prather, 2011).

There are several possible mechanisms for the detritivore reduction of soil N and plant production. The simplest hypothesis is that higher snail activity in the litter and soil of light gaps could cause nitrogen to leach from these highly weathered soils. Although there is little published on the natural history of this species, we anecdotally observed greater snail activity in litter in light gap sites. Alternatively, snails may selectively feed on some important microbial group, altering the functional composition of the litter microbial community, consequently reducing nutrients available for plant growth, a mechanism that has been shown in other ecosystems (Warnock, Fitter, & Usher, 1982), and supported the observation of more fungivores than pure saprophytes in tropical systems (Takeda & Abe, 2001). Furthermore, a companion study examining how these species affected decomposition found no significant effect of snail presence on decomposition rates. This finding suggests that these snails’ effects on litter processes come primarily through microbivory, which may change microbial functional composition, and not comminution (Prather et al., 2018). These two possible mechanisms are not mutually exclusive: Higher snail activity in light gaps with a concomitant reduction of soil N may lead to increases in microbial biomass (Treseder, 2008), giving the microbivores a greater abundance of food.

Although previous literature suggests that tropical forest production is driven by nutrients derived from detritial food webs (Milton & Kaspari, 2007), this study indicates herbivores likely mediated the effects of detritivores (Figure 3) by altering resources reaching detritivores (Prather et al., 2018), by decreasing the quality of litter by increasing the amount of slowly decomposing litter that reached the macrodetritivore community. Herbivore control over detrital food webs has been demonstrated previously (Wardle & Bardgett, 2005), but not in rainforests. Herbivory‐induced shifts to less palatable plant communities have been shown to adversely affect decomposers in other systems (Pastor et al., 1988). Even though nutrients cycled through the detrital food web ultimately provide plant available nutrients, this study suggests that autotrophic food webs may have some control over detrital food web functioning, even in these systems with rapid internal cycling of nutrients.

## 5 CONCLUSIONS: IMPLICATIONS FOR RAINFOREST FUNCTIONING

Consumers from both detrital and autotrophic food webs can affect plant communities in this rainforest, and these effects are likely modified by disturbances. Contrary to the common assumption that detrital food webs control rainforest processes that largely depend upon rapid internal cycling of nutrients, we showed that herbivore mediation of resources reaching detrital food webs might have important consequences for rainforest processes. These results underscore the need for future research examining disturbances and their effects on plant communities and succession to consider the effects of consumer biota, especially in rainforest where consumers are so diverse and numerous (Ellwood & Foster, 2004).

## CONFLICT OF INTEREST

None declared.

## AUTHOR CONTRIBUTIONS

GB and CP conceived of this idea together; CP collected and analyzed data; and CP and GB wrote the manuscript.

## Data Availability

Plant biomass data are available on Figshare (https://doi.org/10.6084/m9.figshare.7571804).
