# Supplementary material for: Herbivore and detritivore effects on rainforest plant production are altered by disturbance
Source: Ecol Evol. 2019 Jun 4;9(13):7652–9. doi: 10.1002/ece3.5316 (PMC6635927; doi:10.1002/ece3.5316)
Supplement: Supplementary file 1 [file ECE3-9-7652-s001.docx]

Electronic Appendix S1.
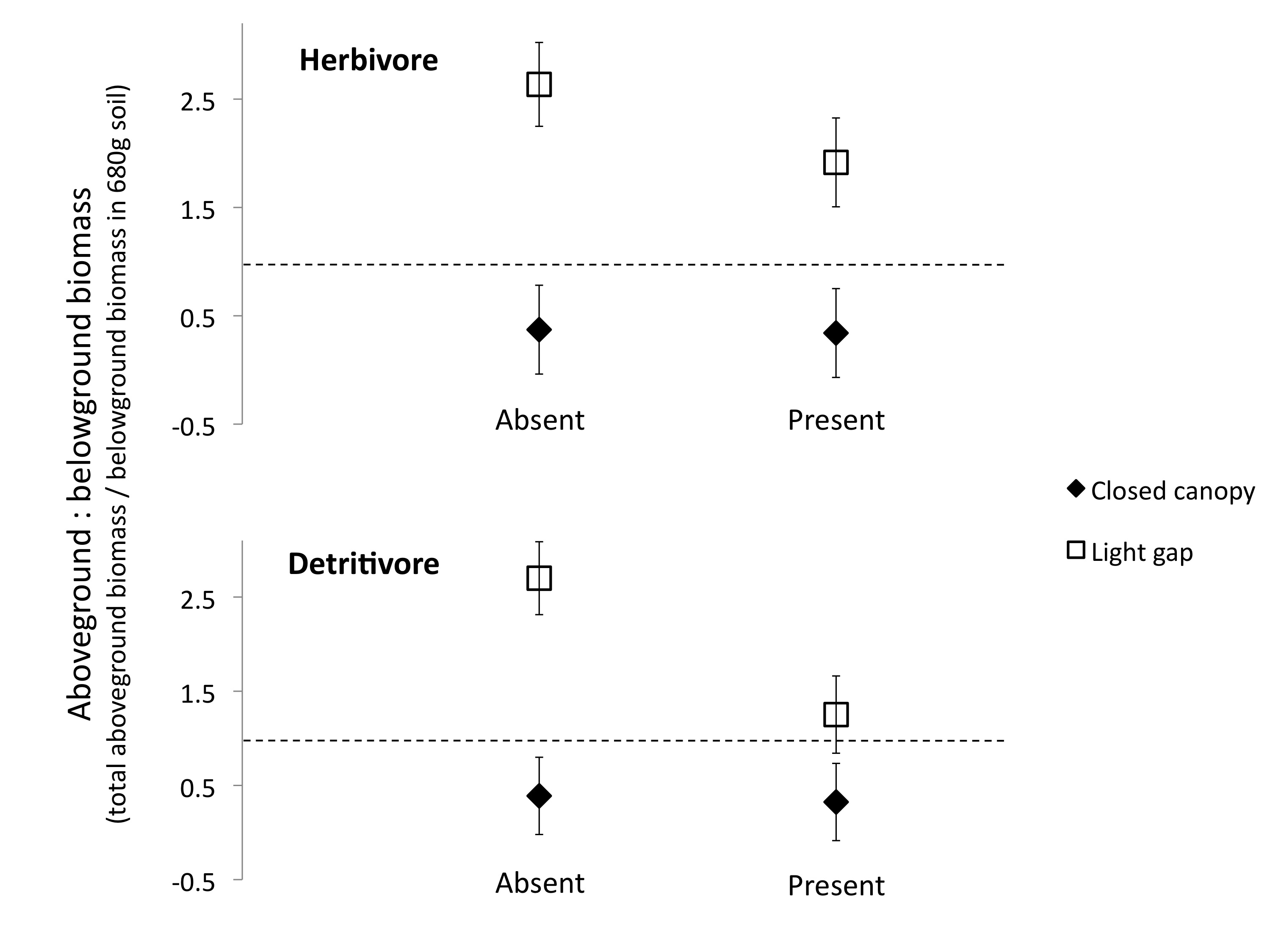


Figure S1. Ratio of aboveground to belowground biomass in A) herbivore and B) detritivore treatments in both light gaps (open squares) and closed canopy sites (black diamonds).

Electronic Appendix S2. Methods for dimensional analysis of plants

The number, length and width of stems, leaves, branches and reproductive parts were measured on each individual plant when they were initially transplanted into enclosures (August, 2005) and annually thereafter in the dry season at LUQ (January of 2006-2008). Plant survival was measured annually and plant abundances were held constant. All plants were harvested at the end of the experiment in August of 2008 and the dimensional variables described above were again measured. Constituent parts (stem, leaves and branches) were separated, dried and weighed to obtain dry biomass that was used to perform dimensional analysis.

Allometric relationships were used to estimate total aboveground biomass at each time point. We used multiple forward linear regressions using one randomly selected individual from each enclosure and control to develop regression models for each plant species. We used plant dimensional variables as independent variables and final aboveground biomass as the dependent variable.

| Species | Regression | df | F | P | Adjusted R^2^ |
| --- | --- | --- | --- | --- | --- |
| *M. prasina* | 0.229*(stem diameter) + 0.36*(height) + 0.562*(number of fruit) + 0.493*(new leaf length) – 0.75*(branch length) | 10, 15 | 4459.99 | <0.001 | 0.766 |
| *P. glabrecens* | 0.329*(stem diameter) + 0.215*(number of new leaves) - 0.359*(number old leaves) + 0.426*(number of branches) + 0.549*(old leaf length) | 11, 16 | 42.59 | <0.001 | 0.642 |
